# Translating Geroscience Into Clinical Practice: Molecular Targets, Emerging Therapies, and Lifestyle Strategies for Healthy Longevity

**DOI:** 10.7759/cureus.117984

**Published:** 2026-10-15

**Authors:** Venkata BharatKumar Pinnelli, Surendra Babu T, Jayashankar C. A., Harsha Anie Mathew, Venkataramana Kandi, Manish G. R., Koshy T Sam, Atul S Sucharitha, Divyadharshini M. S., Satya Vijaya Chandana Batchu, Rakshith C

**Affiliations:** 1 Biochemistry, Vydehi Institute of Medical Sciences and Research Centre, Bengaluru, IND; 2 Anatomy, Vydehi Institute of Medical Sciences and Research Centre, Bengaluru, IND; 3 Internal Medicine, Vydehi Institute of Medical Sciences and Research Centre, Bengaluru, IND; 4 General Medicine, Pushpagiri Institute of Medical Sciences, Thiruvalla, IND; 5 Clinical Microbiology, Infectious Diseases Research Group (IDRG), Squad Medicine and Research (SMR), Amadalavalasa, IND; 6 General Medicine, Akash Institute of Medical Sciences and Research Centre, Bengaluru, IND; 7 General Medicine, Vydehi Institute of Medical Sciences and Research Centre, Bengaluru, IND

**Keywords:** aging, cellular senescence, epigenetic clocks, exercise, geroscience, healthspan, longevity medicine, mtor, nutrition, sleep

## Abstract

Population aging has intensified interest in interventions that preserve function and delay multiple chronic diseases by modifying the biology of aging. The field now spans molecular geroscience, experimental therapeutics, biomarker development, and preventive medicine, but the maturity of evidence varies substantially. This study aimed to synthesize foundational mechanisms, clinical and translational strategies, and lifestyle determinants relevant to healthy longevity, while distinguishing established practice from emerging or unproven interventions. A critical narrative review of PubMed/MEDLINE and reference lists was conducted from January 2000 through August 2026. Priority was given to consensus frameworks, human randomized trials, prospective cohorts, systematic reviews, and influential mechanistic studies. Evidence was interpreted according to study design, clinical relevance, reproducibility, and safety. The expanded 12-hallmark framework depicts aging as an interconnected network involving genomic damage, impaired maintenance systems, maladaptive stress responses, inflammation, and dysbiosis. Senolytics, epigenetic reprogramming, nicotinamide adenine dinucleotide (NAD+) augmentation, mechanistic target of rapamycin (mTOR) inhibition, metformin, and spermidine are biologically plausible, but none yet has definitive evidence of extending lifespan in healthy humans. Epigenetic clocks are useful research biomarkers, although a lower clock estimate is not synonymous with rejuvenation or proven risk reduction. By contrast, cardiorespiratory fitness, muscle strength, dietary quality, restorative and regular sleep, avoidance of tobacco, management of cardiometabolic risk, and social connection are supported by much stronger human-outcome evidence. Responsible longevity medicine should integrate geroscience with rigorous preventive care, use biomarkers as adjuncts rather than diagnoses, and reserve experimental geroprotectors for well-designed trials. The near-term opportunity is not radical life extension, but compression of morbidity through scalable, equitable, and evidence-based preservation of health span.

## Introduction and background

Longevity science asks a clinically important question: can the onset of several age-related diseases be delayed by modifying shared biological processes rather than treating each disease only after it appears? This geroscience hypothesis is distinct from the search for cosmetic youth or indefinite lifespan. Its principal outcome is health span, years lived with preserved physical, cognitive, and social function, and its public-health goal is compression of morbidity. The concept has deep roots in the proposal that the period of disability near the end of life could be compressed, but contemporary demographic experience shows that longer survival does not automatically produce more years in good health [1,2]. Healthy aging must therefore be judged by intrinsic capacity, function, participation, and freedom from disabling disease, not by chronological age alone [3].

Population aging is one of the defining demographic transitions of modern healthcare. Its consequences extend beyond a rising count of older adults: health systems must manage longer periods of multimorbidity, polypharmacy, sensory loss, cognitive impairment, and dependency while preserving autonomy and participation. Globally, individuals aged 60 years and older are projected to double from 1.0 billion in 2020 to 2.1 billion by 2050, with over two-thirds residing in low- and middle-income countries (LMICs) [3]. In rapidly transitioning economies, such as India, the older demographic is expanding at nearly double the rate of the general population, compounding the burden of chronic multimorbidity alongside healthcare constraints [3]. Furthermore, longer survival has not translated into proportional health: contemporary epidemiological estimates document an eight- to 10-year gap between chronological lifespan and disease-free health span [2]. The experience of aging is highly unequal across and within countries because cumulative education, occupation, income, discrimination, environmental exposure, nutrition, and access to prevention shape physiological reserve long before old age [3,4]. A clinically useful longevity framework must therefore address both cellular mechanisms and the life-course conditions that determine who can benefit from prevention. The clinical urgency arises from the clustering of chronic disorders in later life. Cardiovascular disease, cancer, diabetes, neurodegeneration, osteoporosis, and frailty are conventionally managed as separate diagnoses, although they share upstream influences such as impaired repair, metabolic dysregulation, inflammation, and declining physiological reserve.

Geroscience proposes that carefully modifying these shared processes could delay several conditions simultaneously [5]. This approach complements rather than replaces disease-specific prevention, geriatrics, and rehabilitation. Its success would be measured not simply by survival, but by delayed multimorbidity, preserved cognition and mobility, fewer years of dependency, and the ability to participate in personally meaningful activities. This possibility has stimulated a rapidly growing scientific and commercial ecosystem. Academic programs and biotechnology companies are developing senolytics, immune modulators, epigenetic interventions, metabolic drugs, and multi-omic biomarkers, while consumer clinics market biological age tests, supplements, and off-label regimens. Commercial investment can accelerate translation, but it can also reward premature certainty, selective presentation of animal data, and substitution of easily changed biomarkers for outcomes that matter to patients [6].

The professional task is not to reject innovation but to establish evidentiary boundaries that separate plausible research hypotheses from clinically validated care. The field nevertheless faces a substantial evidence gap. Lifespan extension is readily measured in short-lived organisms, whereas a definitive human trial could require decades and expose participants to long-term adverse effects. Researchers therefore rely on intermediate measures, including molecular biomarkers, physiological reserve, incident disease composites, and functional trajectories. Each has limitations. A molecular change may establish that a drug reached its target without demonstrating benefit; improved performance on a short test may not translate into independent living; and observational survival advantages can reflect socioeconomic position, healthier baseline status, or access to care. The challenge is to construct an evidence chain that is biologically coherent, clinically meaningful, and sufficiently sensitive for feasible trials [7,8]. This distinction has become increasingly important as laboratory measures of aging enter clinics and consumer markets. Biological-age algorithms, multi-omic panels, wearable outputs, and imaging-derived scores can stratify risk or illuminate mechanisms, but they differ in what they measure and in how closely they track meaningful outcomes [8-10]. A biomarker may be analytically precise yet clinically unhelpful if it lacks a reference range, is not responsive to intervention, or does not predict benefit when changed. Geroscience consequently requires a translational chain from mechanism to validated biomarker, from biomarker to functional outcome, and ultimately from functional outcome to longer disability-free survival [7]. 

The modern framework began with nine hallmarks of aging and was expanded in 2023 to 12: genomic instability, telomere attrition, epigenetic alterations, loss of proteostasis, disabled macroautophagy, deregulated nutrient sensing, mitochondrial dysfunction, cellular senescence, stem-cell exhaustion, altered intercellular communication, chronic inflammation, and dysbiosis [11]. To provide conceptual clarity for foundational terms used throughout this geroscience framework, operational definitions are summarized in Table 1.

**Table 1:** Working definitions of fundamental geroscience concepts

Concept	Operational definition
Intrinsic capacity	The composite of all physical and mental capacities an individual can draw upon across the life course.
Health span	The period of life spent free from chronic disease, functional disability, and multimorbidity.
Proteostasis	The cellular maintenance network (folding, chaperoning, and degradation) ensuring protein structural integrity.
Cellular senescence	A durable state of cell-cycle arrest with metabolic activity and a secretory phenotype (SASP) driving sterile inflammation.
Senolytics	Pharmacological compounds that selectively induce apoptosis in pro-survival senescent cells.
Epigenetic clocks	Mathematical algorithms predicting biological age or rate of aging from DNA methylation patterns at CpG sites.
Dysbiosis	Pathological perturbation of the composition, diversity, or metabolic function of the gut microbiome.

These processes are neither independent nor uniformly detrimental. Many are adaptive at low intensity or over short periods, yet become harmful when persistent, excessive, or poorly resolved [11]. Consequently, an intervention that improves one laboratory marker may have neutral or adverse effects elsewhere. While prior syntheses have cataloged molecular hallmarks or reviewed lifestyle determinants in isolation, they often blur the distinction between preclinical discoveries and human clinical efficacy or omit translational frameworks linking biomarkers to functional endpoints. The aim of this critical narrative review is to bridge this gap by evaluating foundational mechanisms, emerging pharmacological geroprotectors, and lifestyle determinants against a structured evidentiary hierarchy, thereby defining the translational boundaries of evidence-based longevity practice.

## Review

Methods

Review Design

This article was designed as a critical narrative review integrating molecular geroscience, clinical translation, and lifestyle medicine. A narrative approach was selected because the review questions span heterogeneous interventions, populations, experimental systems, biomarkers, and outcomes that are not suitable for a single pooled effect estimate. The objective was therefore to explain major concepts, identify clinically relevant evidence, and compare the maturity of different longevity strategies rather than to calculate treatment efficacy through meta-analysis. The review was structured around three prespecified domains: foundational and molecular mechanisms; clinical and translational interventions; and lifestyle and preventive determinants of health span.

Information Sources and Search Strategy

Targeted searches of PubMed/MEDLINE were conducted from January 1, 2000, through August 23, 2026, restricted to peer-reviewed, English-language publications. Database searches were supplemented by backward citation tracking of seminal consensus frameworks, guidelines, systematic reviews, and landmark clinical trials. Titles, abstracts, and full texts were screened by two independent reviewers, with disagreements resolved by consensus discussion with a third senior reviewer. The search concept combined aging and geroscience terminology with intervention-specific keywords linked via explicit Boolean operators (AND, OR), as detailed in Table 2.

**Table 2:** Narrative search strategy and database query specifications AMPK: adenosine monophosphate-activated protein kinase; mTOR: mechanistic target of rapamycin; NAD+: nicotinamide adenine dinucleotide; VO_2_max: maximal oxygen consumption

Database/source	Search terms and Boolean combinations	Target field/limits	Search date window
PubMed/MEDLINE	("aging" OR "ageing" OR "longevity" OR "health span" OR "geroscience") AND ("hallmarks of aging" OR "cellular senescence" OR "senolytic" OR "epigenetic clock" OR "Horvath" OR "GrimAge" OR "DunedinPACE" OR "mitochondrial dysfunction" OR "mitophagy" OR "nicotinamide adenine dinucleotide" OR "NAD+") AND ("longevity medicine" OR "rapamycin" OR "metformin" OR "spermidine" OR "mechanistic target of rapamycin" OR "mTOR" OR "AMPK" OR "sirtuin" OR "gut microbiome")	Title, abstract, MeSH terms; English language	January 1, 2000-August 23, 2026
	("aging" OR "ageing" OR "longevity" OR "health span" OR "geroscience") AND ("cardiorespiratory fitness" OR "VO_2_max" OR "Zone 2" OR "resistance training" OR "caloric restriction" OR "intermittent fasting" OR "time-restricted eating" OR "sleep" OR "circadian rhythm" OR "stress" OR "social isolation" OR "centenarian" OR "Blue Zones")	Combined with filters/keywords for randomized trials, cohort studies, systematic reviews, meta-analyses, mortality, morbidity, physical function, cognition, safety, and quality of life	January 1, 2000-August 23, 2026
Reference lists/manual searching	Backward citation tracking of landmark clinical trials, consensus frameworks, guideline reports, and systematic reviews	Full-text reviews and seminal foundational papers	Through August 23, 2026

Time Frame and Eligibility Criteria

The principal evidence window was January 2000 through August 2026, reflecting the rapid development of modern geroscience. Earlier publications were retained when they introduced foundational concepts, validated long-lived populations, or established influential clinical and epidemiological observations. Eligible sources were peer-reviewed English-language articles relevant to aging mechanisms, human health span, functional aging, age-related disease, or the safety and effectiveness of candidate interventions. Priority was assigned to consensus and framework papers, randomized controlled trials, prospective cohorts, systematic reviews, meta-analyses, and well-characterized human biomarker studies. Preclinical studies were included selectively when they established causal plausibility, demonstrated lifespan or health span effects, or clarified mechanisms that could not yet be evaluated directly in humans. Comprehensive inclusion and exclusion criteria applied during study selection are summarized in Table 3.

**Table 3:** Eligibility criteria for evidence selection

Criterion category	Inclusion criteria	Exclusion criteria
Study design and evidence type	Consensus and framework statements, randomized controlled trials, prospective cohort studies, systematic reviews, meta-analyses, and well-characterized human biomarker studies	Conference abstracts, isolated case reports, non-peer-reviewed promotional materials, and speculative commentaries
Preclinical and mechanistic research	Preclinical studies establishing direct causal plausibility, demonstrating lifespan/healthspan effects, or clarifying mechanisms not directly testable in humans	Studies lacking mechanistic relevance to aging biology, cellular maintenance, or human healthspan
Outcomes and clinical relevance	Functional aging, intrinsic capacity, physiological reserve, age-related chronic disease composites, disability-free survival, morbidity, and mortality	Studies focusing exclusively on cosmetic outcomes or aesthetic aging
Therapeutic and protocol validity	Interventions with transparent methodological descriptions, verifiable dosing, and defined safety or target-engagement profiles	Studies evaluating unsupported commercial protocols or making therapeutic claims without adequate methodological information
Disease-specific populations	Studies of clinical populations providing critical safety, pharmacodynamic, or proof-of-concept translational data	Studies assuming disease-specific therapeutic responses generalize directly to healthy adults without qualification
Language and publication scope	Peer-reviewed articles published in the English language.	Non-English publications and non-peer-reviewed white papers or grey literature.
Publication timeframe	Primary evidence window from January 2000 through August 2026, supplemented by earlier landmark historical sources.	Outdated commentaries or non-validated historical cohorts lacking demographic rigor.

Study Selection and Evidence Appraisal

Titles, abstracts, and available full texts were assessed for relevance to the prespecified domains. When several publications addressed the same question, preference was given to the most methodologically informative or recent synthesis, supplemented by landmark primary studies. Evidence 4 was interpreted according to study design, sample size, population, comparator, duration, endpoint validity, consistency, effect magnitude, and adverse-event reporting. Particular attention was paid to the distinction between association and causation, animal lifespan extension and human benefit, biochemical target engagement and functional improvement, and exploratory biomarker change and patient-important outcomes. This critical narrative review was structured in accordance with the SANRA (Scale for the Assessment of Narrative Review Articles) criteria, with evidence appraised against a defined hierarchy that prioritized multicenter randomized controlled trials and systematic reviews addressing hard clinical endpoints such as mortality, incident chronic disease, and validated measures of physical or cognitive function; followed by well-characterized prospective cohort studies reporting multivariable-adjusted risk estimates; early-phase human trials demonstrating target engagement, safety, or surrogate biomarker modulation; and, lastly, preclinical vertebrate mammalian models and in vitro mechanistic studies. Findings from observational studies were interpreted cautiously because of confounding, reverse causation, healthy user effects, and survivor bias. Small uncontrolled intervention studies were treated as hypothesis-generating, even when statistically significant changes in biological-age measures or molecular markers were reported.

Data Synthesis and Methodological Limitations

Evidence was synthesized qualitatively by comparing biological rationale, human evidence, safety, and clinical readiness across interventions. No quantitative pooling was attempted because exposures, doses, populations, follow-up periods, and outcomes were highly heterogeneous. The review did not use duplicate independent screening, formal study-level risk-of-bias instruments, or certainty grading, and no protocol was registered. It should therefore be interpreted as a structured critical overview rather than a systematic review. These limitations were addressed by prioritizing higher-level human evidence, explicitly identifying translational gaps, and avoiding clinical recommendations based solely on animal findings, surrogate outcomes, or single exploratory studies.

Foundational and Molecular Science

The 12 hallmarks - from mechanisms to therapeutic targets: The hallmarks form a layered network rather than a checklist. Primary damage includes genomic instability, telomere attrition, epigenetic alterations, and loss of proteostasis. Antagonistic responses include nutrient-sensing changes, mitochondrial stress, senescence, and altered autophagy; these can initially preserve survival but become pathogenic when chronic. Integrative consequences include stem-cell exhaustion, altered intercellular communication, inflammation, and dysbiosis [11]. Deoxyribonucleic acid (DNA) damage can induce senescence; senescent-cell secretions can drive inflammation; inflammation can impair stem cells and mitochondrial function; and dysbiosis can amplify immune and metabolic disturbance. This interdependence makes single-target “reversal” claims biologically improbable (Figure 1).

**Figure 1:**
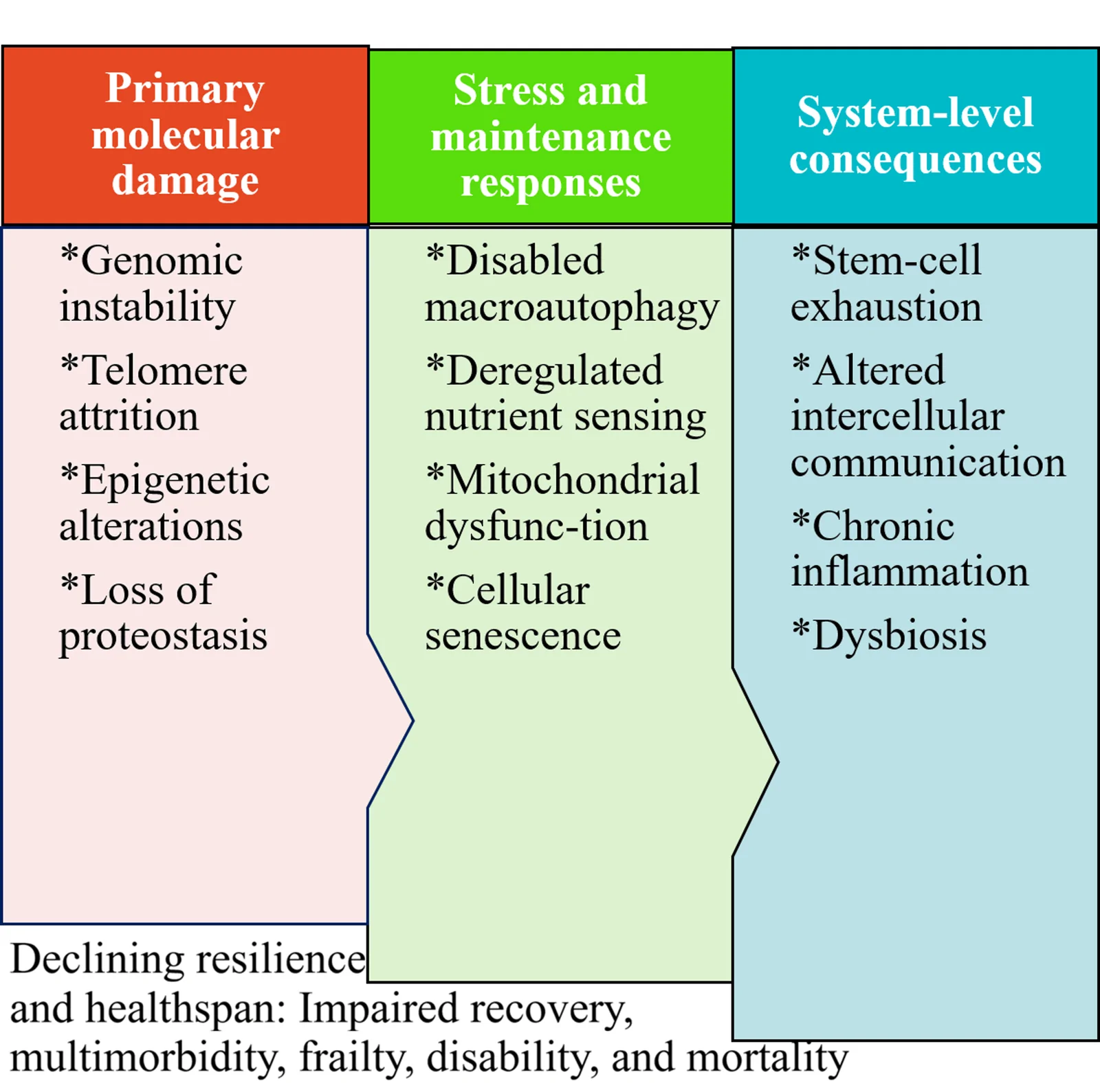
Integrated representation of the 12 hallmarks of aging This image has been synthesized from Reference [11]. Image credit: This is an original image created by the authors using Microsoft Word/PowerPoint (Microsoft® Corp., Redmond, WA).

The framework generates testable targets, but a hallmark is not automatically a validated drug target. Causal evidence is strongest when aggravation accelerates dysfunction, and selective correction delays it. Even then, timing, tissue, dose, sex, genotype, and disease context matter: processes, such as autophagy and inflammation, can be protective or harmful depending on context. Translation should therefore seek calibrated restoration of homeostasis rather than maximal pathway activation or suppression, progressing across defined evidentiary stages from target validation to clinical implementation [11] (Figure 2).

**Figure 2:**
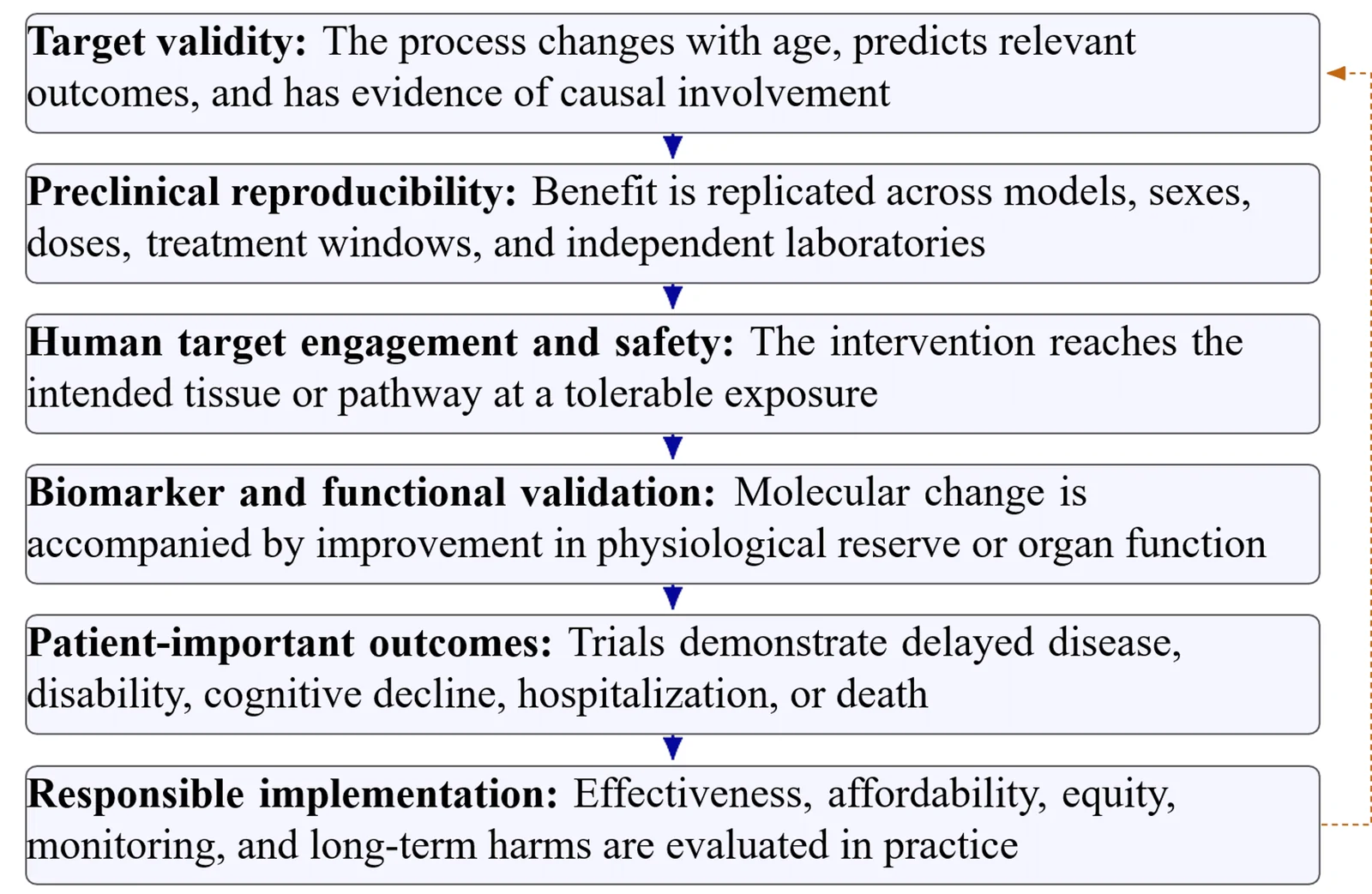
Translational pathway for a candidate longevity intervention This image has been synthesized from References [7,8]. Image credit: This is an original image created by the authors using Microsoft Word/PowerPoint (Microsoft® Corp., Redmond, WA).

Cellular Senescence and Senolytics

Cellular senescence is a durable stress response characterized by proliferative arrest, altered metabolism, resistance to apoptosis, chromatin remodeling, and, in many cells, a senescence-associated secretory phenotype (SASP). No single marker identifies all senescent cells, and senescence has beneficial roles in development, wound repair, and tumor suppression [12,13]. With age, however, inefficient immune clearance permits some senescent populations to accumulate. Their cytokines, chemokines, proteases, and growth factors can disrupt tissue structure, propagate secondary senescence, and sustain sterile inflammation. Genetic clearance experiments and senolytic studies in mice support a causal contribution to multimorbidity, physical dysfunction, and shortened survival [14,15]. Senolytics exploit pro-survival dependencies of senescent cells; examples include dasatinib plus quercetin, fisetin, and B-cell lymphoma 2 (BCL-2)-family inhibitors. Senomorphics suppress harmful features, such as the SASP, without killing the cell. Early human studies show target engagement in small disease-specific cohorts, but their size, controls, endpoints, and follow-up cannot establish broad benefit [16,17]. Cytopenias, infection, bleeding, interactions, impaired healing, and loss of beneficial senescent cells remain concerns. “Zombie cell” is a metaphor, not a justification for self-treatment.

Although early human trials confirm target engagement and safety in disease-specific cohorts (such as diabetic kidney disease and idiopathic pulmonary fibrosis), several broader phase 2 trials evaluating dasatinib plus quercetin and fisetin remain active or have yet to report functional outcomes; claims of systemic rejuvenation remain unverified.

Epigenetic Clocks and Claims of Biological-Age Reversal

DNA-methylation clocks compress methylation at selected cytosine-phosphate-guanine (CpG) sites into age-related measures. The original Horvath multi-tissue clock was trained to predict chronological age [18]. Second-generation measures were optimized for health outcomes: GrimAge incorporates methylation surrogates for smoking and plasma proteins and predicts morbidity and mortality, while Dunedin (P)ace of (A)ging (C)alculated from the (E)pigenome (DunedinPACE) estimates the current pace of multisystem biological decline rather than accumulated years [19,20]. These tools answer different questions and should not be used interchangeably (Table 4).

**Table 4:** Conceptual comparison of commonly used DNA‑methylation aging measures DunedinPACE: Dunedin (P)ace of (A)ging (C)alculated from the (E)pigenome

Measure	Primary construct	Potential research use	Important limitation
Horvath clock [18]	Chronological‑age prediction across multiple tissues	Quantifies deviation from expected methylation age; supports cross‑tissue aging research	Not trained primarily on morbidity, mortality, or intervention response
PhenoAge [21]	Methylation surrogate of a mortality‑related clinical phenotype	Links methylation patterns to phenotypic risk and age‑related disease burden	Can respond differently from other clocks; lower values do not prove rejuvenation
GrimAge [19]	Methylation surrogates for smoking history and selected plasma proteins	Strong risk stratification for mortality and several age‑related outcomes	Sensitive to exposure history; not a direct measure of every tissue’s aging state
DunedinPACE [20]	Current pace of multisystem biological change	Evaluates whether aging is proceeding faster or slower; may be useful in intervention studies	Reports a rate rather than accumulated biological age; clinical thresholds remain unsettled

Clock acceleration is associated with adverse outcomes, but clinical use is constrained. PhenoAge illustrates how a clock’s training target shapes its interpretation [21]. However, because estimates vary across tissues, platforms, and ancestries, longitudinal validation is essential before inferring clinical mediation [22]. In Comprehensive Assessment of Long-Term Effects of Reducing Intake of Energy (CALERIE), caloric restriction modestly slowed DunedinPACE but did not significantly change GrimAge or PhenoAge, illustrating discordance among measures [23]. The Thymus Regeneration, Immunorestoration, and Insulin Mitigation (TRIIM) pilot reported younger methylation-age estimates after a one-year thymic-regeneration regimen, but it included only nine men and lacked a randomized control group [24]. Partial cellular reprogramming can restore selected molecular and functional features in mice, yet loss of cell identity, organ toxicity, and tumorigenesis remain major translational hazards [25]. Thus, “biological age reversal” should be reserved for convergent improvement across validated biomarkers, organ function, and durable clinical outcomes, not a favorable change in one commercial test (Figure 3).

**Figure 3:**
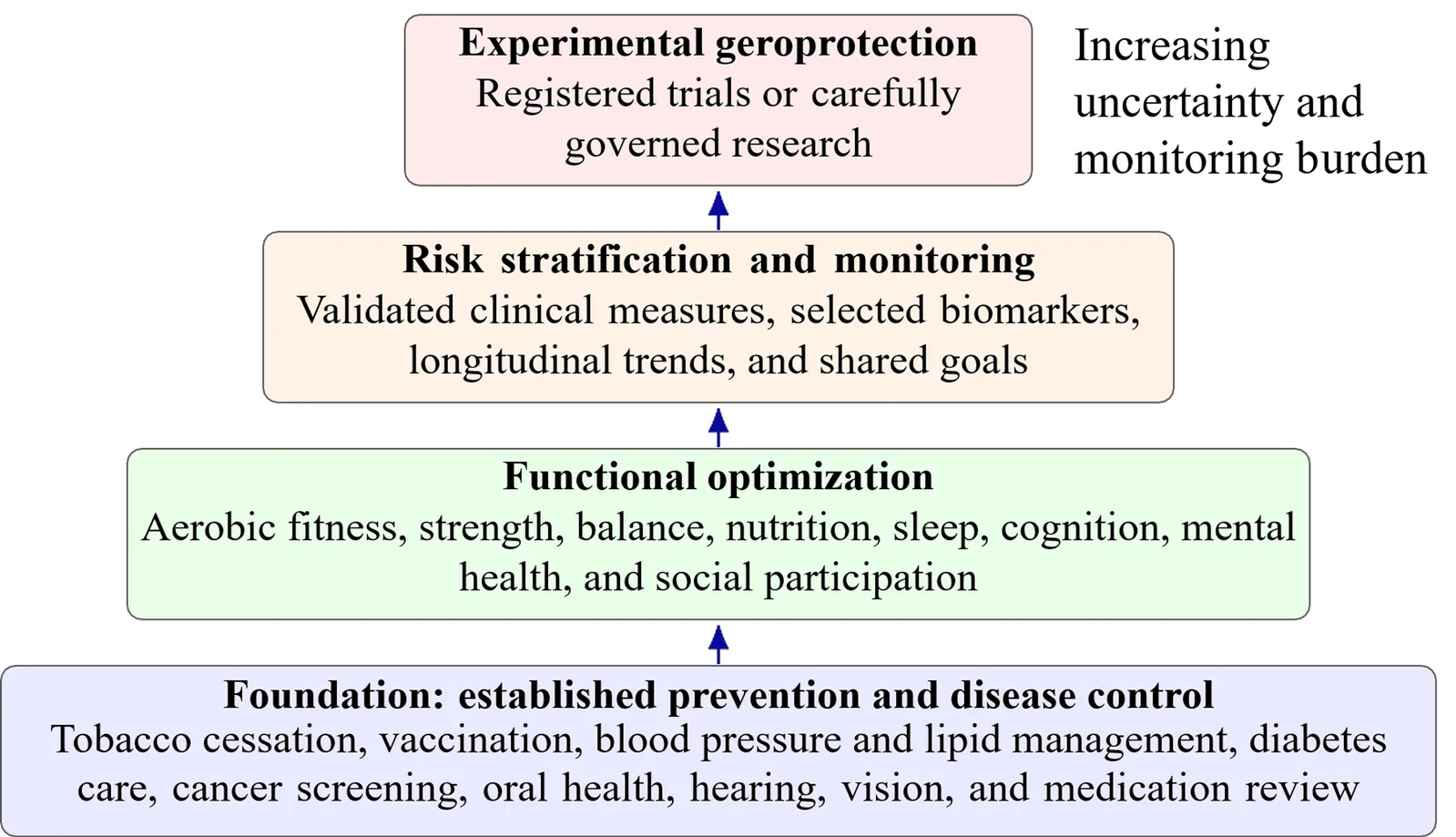
Layered model of responsible longevity medicine This image has been synthesized from references [3,7,10]. Image credit: This is an original image created by the authors using Microsoft Word/PowerPoint (Microsoft® Corp., Redmond, WA).

Clinical Assessment, Biomarkers, and Trial Endpoints

The clinical translation of geroscience depends on measurement, but not every measurable feature is a useful biomarker. A clinically credible marker should have analytical validity, reproducibility, a defined reference context, biological relevance, and an interpretation that changes management. It should also add information beyond inexpensive conventional measures. Biomarkers can be organized into several categories: exposures and behaviors; established disease risk factors; organ-specific reserve; systemic functional measures; and putative measures of aging biology, including inflammatory signatures, proteomic or metabolomic scores, and epigenetic clocks. These categories should not be collapsed into a single “biological age” because they represent different causal pathways and time scales [7-9]. Serial measurement can be more informative than a single result, but longitudinal testing introduces regression to the mean, biological variability, laboratory drift, and behavioral changes caused by awareness of testing. A meaningful response should therefore be prespecified and interpreted alongside function and safety. For example, a favorable change in a methylation clock is more persuasive if accompanied by improved physiological reserve, whereas a change without functional benefit remains exploratory. Similarly, increases in circulating NAD-related metabolites demonstrate exposure to a precursor but do not establish improved mitochondrial function or slower aging [22,23,26-29]. Clinical trials face a related endpoint problem. Mortality alone is definitive but inefficient, and testing one disease at a time may miss a multisystem effect. Composite outcomes can combine incident cardiovascular disease, cancer, diabetes, dementia, and death, while complementary measures capture mobility, cognition, frailty, hospitalization, and quality of life. Such composites require careful adjudication because common but mild events can dominate more serious outcomes. Trials must also measure harms with the same rigor as benefits, particularly when treating generally healthy people who have a low tolerance for toxicity [7,30]. The translational standard is strongest when an intervention demonstrates target engagement, changes a validated intermediate phenotype, preserves function, and reduces patient-important events without unacceptable harm. This staged approach is slower than direct-to-consumer adoption but protects patients from interventions that improve a laboratory score while worsening another organ system or adding treatment burden [7,8].

Patient heterogeneity adds another translational layer to clinical assessment. The balance between growth and repair, immune activation and suppression, or energy restriction and adequate nutrition differs substantially across a healthy middle-aged adult, an older adult with obesity, and a frail individual at risk of sarcopenia. Interventions may also vary by sex, genotype, medication exposure, microbiome, social conditions, and baseline fitness. Precision longevity medicine should therefore prioritize selecting an intervention for a defined biological and clinical context, supported by explicit monitoring and stopping rules, rather than applying uniform supplement regimens, fasting schedules, biomarker targets, or pharmaceuticals across diverse populations [3,10].

Rapamycin, Metformin, and Spermidine

Rapamycin inhibits mechanistic target of rapamycin (mTOR) complex 1 (mTORC1), promotes autophagy, and extends lifespan across several model organisms, including genetically heterogeneous mice treated late in life [31]. A 2025 vertebrate meta-analysis found a significant lifespan effect for rapamycin but not metformin, while also identifying substantial heterogeneity and publication bias [32]. In older adults, low-dose rapalogs have improved influenza-vaccine responses and some measures of antiviral immunity, but a later program produced mixed results for respiratory outcomes [33,34]. Small trials suggest that intermittent low-dose rapamycin can be feasible, yet they are underpowered for uncommon or long-latency harms [35]. Stomatitis, dyslipidemia, edema, impaired glucose control, infection, pneumonitis, wound complications, and drug interactions require attention. Evidence does not support routine prescribing solely to extend lifespan in healthy people. Translation is complicated because “mTOR inhibition” is not a single exposure. Dose, duration, tissue penetration, treatment interval, and relative effects on mTORC1 and mTOR complex 2 (mTORC2) can alter both efficacy and toxicity. Transplant regimens were designed for immunosuppression, whereas geroscience studies generally seek partial or intermittent mTORC1 inhibition that preserves immune competence and tissue repair. In a placebo-controlled study, a TORC1 inhibitor enhanced selected immune responses and reduced reported infections, supporting target engagement but not demonstrating slower aging or longer life [36]. Future trials need to establish whether immune, cardiovascular, cognitive, or physical-function signals are reproducible and whether benefits persist after treatment stops. Safety interpretation must reflect the preventive setting. An adverse-event profile acceptable for organ transplantation or cancer may be unacceptable for a healthy adult seeking risk reduction. Research protocols therefore require explicit exclusion criteria, interaction review, vaccination and infection planning, laboratory surveillance, and stopping rules. Rare harms and potential effects on wound healing, glucose regulation, fertility, and vaccine response may require larger and longer studies than existing trials. The central uncertainty is not whether rapamycin engages a conserved aging pathway, but whether a clinically useful therapeutic window exists for heterogeneous humans [34,35,37].

Metformin activates AMPK indirectly through energetic stress, reduces hepatic glucose production, and has decades of safety data in diabetes. Observational comparisons have motivated the Targeting Aging With Metformin (TAME) concept, which tests whether a composite of age-related clinical events can be delayed [30,38,39]. However, confounding by indication and comparator choice limit observational longevity claims. Gastrointestinal effects, vitamin B12 deficiency, renal dosing constraints, and rare lactic acidosis are established risks. In one randomized study, metformin blunted some mitochondrial and cardiorespiratory adaptations to aerobic training in older adults, emphasizing that drug-exercise interactions may vary between individuals [40]. Metformin is evidence-based for appropriate metabolic indications, not yet as a universal geroprotector. Metformin demonstrates how established safety in diabetes can be prematurely conflated with efficacy in healthy aging. Treatment response depends heavily on baseline insulin sensitivity, nutritional status, and concurrent exercise. While the TAME trial establishes a regulatory framework by targeting a composite of major age-related clinical events, definitive prospective trial readouts in non-diabetic populations remain pending [30,38].

Spermidine is a dietary polyamine involved in autophagy, translation, mitochondrial function, and chromatin regulation. Lifespan extension is reproducible in several model systems, and observational studies link spermidine-rich diets with favorable outcomes [41]. In the adequately powered SmartAge randomized trial, 12 months of supplementation did not significantly improve the primary memory outcome in older adults with subjective cognitive decline, despite earlier pilot signals [42]. This pattern, including strong mechanism, encouraging observational data, and a neutral primary trial, illustrates why clinical recommendations must follow, not precede, confirmatory evidence. Dietary exposure is further confounded by overall nutritional patterns and variable supplement purity. Given the neutral primary cognitive outcome in SmartAge, secondary mechanistic signals must not be interpreted as clinical rejuvenation; future trials must establish whether targeted autophagy induction translates into verifiable cognitive or physical preservation [41,42].

mTOR, AMPK, and Sirtuins as a Nutrient-Sensing Network

mTOR promotes growth and protein synthesis when nutrients and growth factors are abundant. AMPK detects low cellular energy and supports catabolism, mitochondrial biogenesis, and autophagy. Sirtuins are nicotinamide adenine dinucleotide (NAD+)-dependent deacylases that coordinate stress resistance, metabolism, DNA repair, and circadian programs [37,43,44]. These pathways cross-regulate one another: AMPK can restrain mTORC1, NAD+ availability influences sirtuins, and both fasting and exercise produce time-dependent signaling across the network. No pathway should be permanently “on” or “off.” Chronic mTOR suppression may impair immunity and repair, persistent AMPK activation may be undesirable in frailty, and nonspecific sirtuin activators have disappointed clinically. Health span probably depends on flexible switching between feeding and fasting, activity and recovery, and anabolism and maintenance [37,43,44]. This network helps explain why physiological interventions can have broad effects without acting as simple drug mimetics. Feeding and resistance exercise generate transient anabolic signaling needed for muscle remodeling, while fasting and endurance activity promote energetic stress responses and quality control. In older adults, excessive suppression of growth signaling could worsen anabolic resistance, sarcopenia, or recovery even if it improves selected molecular markers. Conversely, chronic nutrient excess can maintain mTORC1 signaling and impair metabolic flexibility. The desired state is rhythmic responsiveness rather than permanent pathway inhibition [37,43]. Tissue specificity is another barrier to translation. AMPK activation that benefits liver glucose handling may have different implications in skeletal muscle, immune cells, or the hypothalamus. Sirtuin activity depends on cellular NAD+ pools that are compartmentalized and cannot be inferred fully from a blood measurement. mTORC1 inhibition may promote autophagy in one tissue while impairing wound healing or immune responses elsewhere. Accordingly, future drugs may need tissue targeting, intermittent delivery, or biomarker-defined selection rather than systemic continuous exposure [27,37,44].

The Gut Microbiome and Longevity

The gut microbiome influences barrier integrity, immune education, bile-acid metabolism, short-chain fatty-acid production, and drug response. Aging is often associated with reduced resilience and altered community structure, but there is no universal “young” or “healthy” microbiome. Geography, medications, diet, frailty, institutionalization, stool transit, and sequencing methods can outweigh chronological age [45,46]. Centenarian studies have reported distinctive community structures and enrichment of organisms capable of producing unusual secondary bile acids, while a large cohort found that increasing microbiome uniqueness in later life predicted survival [47-49]. These findings are intriguing but remain susceptible to survivor, geographic, dietary, and cohort effects. The most defensible clinical strategy is to support microbial function through dietary diversity, 12 fermentable fiber, plant foods, physical activity, and judicious antibiotic use. In the New Dietary Strategies Addressing the Specific Needs of Elderly Population for a Healthy Ageing in Europe (NU-AGE) intervention, Mediterranean-diet adherence altered microbial features associated with lower frailty and inflammation, although mediation of clinical benefit remains difficult to prove [50].

Probiotics have strain- and indication-specific effects; commercial stool scores often lack validated therapeutic thresholds; and fecal microbiota transplantation should remain restricted to established indications or trials. The microbiome is a promising mediator and biomarker, but causal longevity interventions require standardized sampling, functional readouts, and randomized trials with clinical endpoints. The central challenge is distinguishing a microbial signature of healthy behavior from a causal driver of healthy aging. People who remain independent into advanced age differ in diet, medication exposure, residence, mobility, and healthcare contact, all of which shape the microbiome. Taxonomic abundance alone also provides an incomplete picture because different organisms can perform similar metabolic functions. Metagenomics, metabolomics, bile-acid profiling, and repeated sampling may be more informative than a single commercial diversity score [45,46,49].

Intervention design should therefore focus on defined functions and clinical outcomes. A probiotic must be evaluated at the strain level, a prebiotic must specify its substrate and target population, and a dietary intervention should account for baseline diet and adherence. Fecal microbiota transplantation can transfer complex communities but also introduces infection, donor-selection, durability, and regulatory concerns. For general longevity care, a varied fiber-rich dietary pattern remains more defensible than attempting to engineer a universally “young” microbiome. Future trials should test whether microbial changes mediate improvements in frailty, cognition, metabolic health, or infection risk rather than treating compositional change as an endpoint in itself [46,50].

Lifestyle and Preventive Medicine

Exercise, nutrition, sleep and circadian alignment, and psychosocial health influence overlapping biological pathways rather than acting as independent prescriptions. Figure 4 summarizes their convergence on shared mediators of functional aging [51-54].

**Figure 4:**
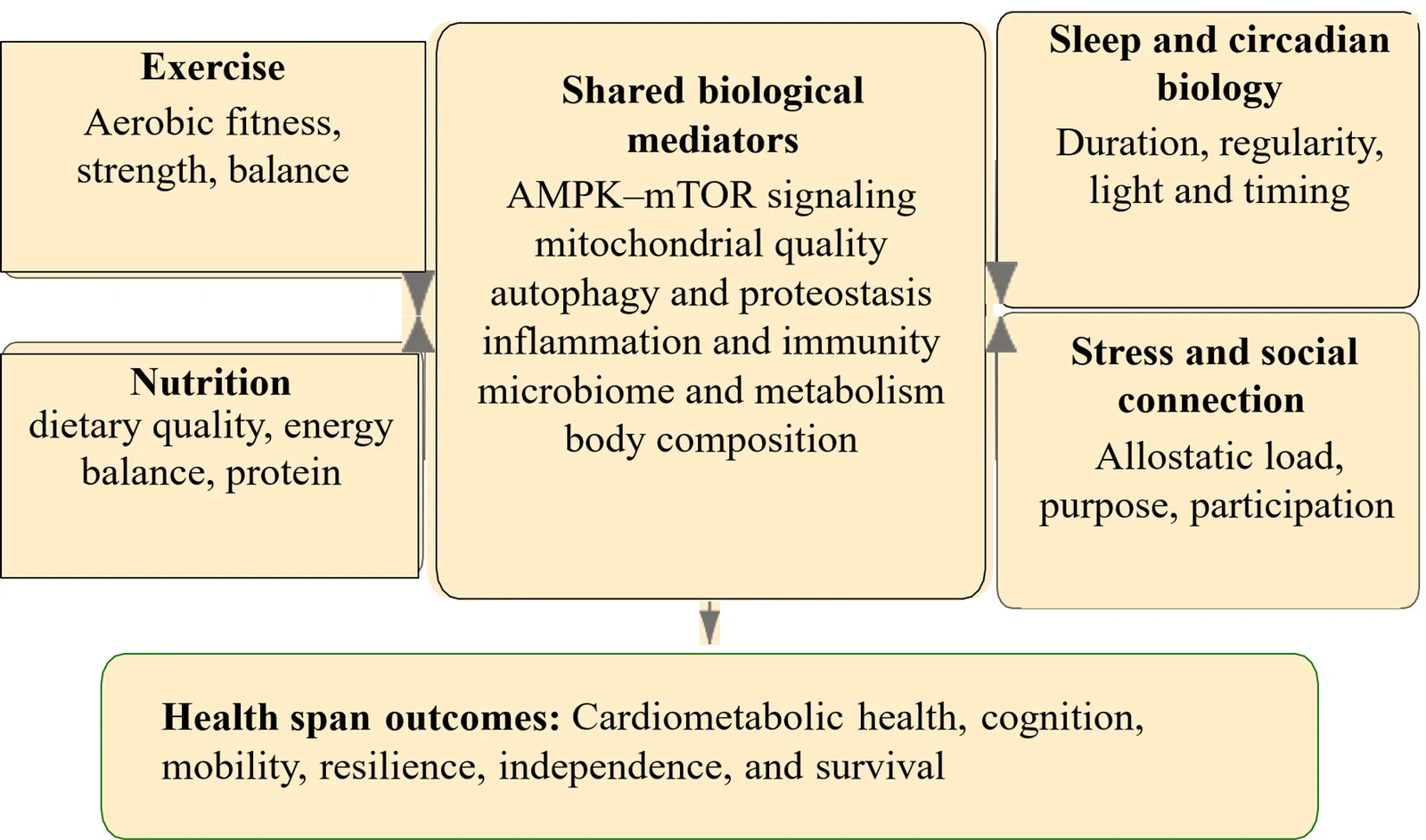
Interacting lifestyle determinants of longevity This image has been synthesized from References [51-54]. AMPK: adenosine monophosphate-activated protein kinase; mTOR: mechanistic target of rapamycin Image credit: This is an original image created by the authors using Microsoft Word/PowerPoint (Microsoft® Corp., Redmond, WA).

Exercise as a longevity drug: Zone 2, VO_2_max, and muscle: Exercise is the closest available intervention to a broad-spectrum geroprotector because it improves cardiorespiratory, metabolic, vascular, musculoskeletal, immune, and neurocognitive function. Objectively measured cardiorespiratory fitness shows a strong graded inverse association with mortality across age, sex, and racial groups [55,56]. Accelerometer studies likewise show that replacing sedentary time with activity is associated with lower mortality, with the largest proportional gains often occurring when inactive people begin moving [57]. Fitness is not merely a risk marker: training increases stroke volume, capillary density, mitochondrial capacity, insulin sensitivity, and autonomic regulation. “Zone 2” usually means sustained work below the first lactate or ventilatory threshold, although heart-rate systems are not equivalent [58]. It allows aerobic volume with manageable fatigue, but its adaptations are not unique, and it has no direct lifespan evidence. Complete programs also include some vigorous work to raise VO2max and frequent low-intensity movement to reduce sedentary time.

Skeletal muscle is a metabolic reservoir and an endocrine organ that releases myokines and buffers glucose and amino acids. Low strength predicts mortality, while muscle-strengthening activity is associated with lower risks of death and major chronic disease [59,60]. Resistance, power, balance, and impact-bearing exercise preserve independence, bone, and fall resistance. A pragmatic longevity prescription therefore combines at least 150 minutes per week of moderate aerobic activity (or an equivalent vigorous dose), resistance training on at least two days, balance work for those at risk, and progressive overload individualized to disease, frailty, and recovery [51]. Large cohort data suggest benefit across a wide range of moderate and vigorous activity volumes rather than a single optimal threshold (Table 5) [61].

**Table 5:** Selected longevity interventions and present evidentiary maturity AMPK: adenosine monophosphate-activated protein kinase; mTOR: mechanistic target of rapamycin; mTORC1: mechanistic target of rapamycin complex 1; NAD+: nicotinamide adenine dinucleotide; RCT: randomized controlled trial

Intervention	Primary target	Human evidence	Current interpretation
Senolytics [16,17]	Senescent-cell survival pathways	Small, short, disease-specific trials demonstrate feasibility and some target engagement	Experimental; benefit-risk and target-cell specificity unresolved
Rapamycin and rapalogs [33-36]	mTORC1 and autophagy	Immune-response signals and mixed respiratory outcomes; no lifespan trial	Do not use routinely for healthy longevity outside research
Metformin [30,38-40]	Hepatic metabolism, AMPK-linked signaling	Strong diabetes evidence; longevity inference mainly observational; aging-outcome trial concept established	Use for recognized indications; geroprotection unproven
Spermidine [41,42]	Polyamine metabolism and autophagy	Mechanistic and observational support; neutral primary cognitive endpoint in a larger RCT	Food sources reasonable; supplements not established geroprotectors
NAD+ precursors [27-29]	NAD+ availability, sirtuins, repair pathways	Biochemical target engagement; inconsistent functional outcomes in small trials	Long-term clinical value and safety remain uncertain
Exercise [51,55-57,59-61]	Multisystem adaptation	Consistent cohorts, trials of intermediate outcomes, and dose-response associations with mortality and disability	Core standard of preventive longevity care
Dietary quality [50,52]	Cardiometabolic risk, inflammation, microbiome	Randomized cardiovascular evidence and extensive cohort data	Prefer sustainable, minimally processed, plant-forward patterns
Sleep regularity and adequacy [53]	Circadian and homeostatic regulation	Strong observational outcome evidence; effective treatment available for major sleep disorders	Assess routinely and treat clinically significant disorders

Caloric Restriction, Intermittent Fasting, and Longevity Diets

Caloric restriction extends lifespan in many model organisms, partly through nutrient sensing, autophagy, metabolic efficiency, and reduced growth signaling. In CALERIE, adults without obesity achieved modest sustained restriction over two years, with improvements in several cardiometabolic measures, evidence of metabolic slowing and reduced oxidative stress, and a small slowing of DunedinPACE [23,62]. The study did not demonstrate longer life, and chronic restriction can cause hunger, cold intolerance, loss of lean mass or bone, menstrual disturbance, and reduced quality of life. It is inappropriate for underweight people, pregnancy, eating disorders, many frail older adults, or without attention to protein, micronutrients, and resistance exercise. Intermittent fasting and time-restricted eating can simplify energy restriction and align food intake with circadian metabolism [63]. Small controlled studies of early time-restricted feeding show improvements in insulin sensitivity even without weight loss, but larger trials demonstrate that results depend on comparator, timing, adherence, and energy intake [64,65]. In adults with obesity, adding an eight-hour eating window to caloric restriction did not produce greater 12-month weight loss than caloric restriction alone [66]. Long-term evidence for prevention of disability, dementia, or death is lacking. Earlier daytime intake is generally more concordant with circadian insulin sensitivity than late-night eating, but adherence and clinical context take priority. The strongest dietary evidence supports patterns rather than a single macronutrient ratio: vegetables, fruit, legumes, whole grains, nuts, seeds, unsaturated fats, and adequate high-quality protein; limited refined grains, added sugars, processed meat, and ultra-processed foods; and energy intake matched to healthy body composition. The Mediterranean dietary pattern reduces major cardiovascular events in high-risk adults [52]. For older adults, avoiding sarcopenia may be more important than aggressive leanness. A “longevity diet” should therefore be culturally acceptable, affordable, fiber-rich, minimally processed, and sufficient in protein and energy.

Sleep, Circadian Biology, and Accelerated Aging

Sleep supports memory consolidation, immune regulation, glucose control, vascular health, and proteostatic clearance. Adults should generally obtain at least seven hours regularly, while individual need and older-adult sleep architecture vary [53]. Both short and long sleep are associated with mortality, although long sleep may reflect underlying disease. Objective cohort data suggest that day-to-day regularity of sleep timing predicts mortality at least as strongly as duration [67]. Circadian disruption uncouples central and peripheral clocks that organize metabolism, hormone secretion, immune activity, and cellular repair; aging itself can weaken the amplitude and synchrony of these rhythms [68]. Short or fragmented sleep and insomnia symptoms are associated with faster GrimAge and pace-of-aging measures, but causality and reversibility are not fully established [69]. Meta-analytic evidence also links both unusually short and long reported sleep with mortality, although reverse causation is important [70]. Clinically, the priority is not a wearable’s proprietary sleep score. It is a regular sleep-wake schedule, morning light, daytime activity, reduced bright light and heavy meals late at night, and diagnosis of treatable disorders such as obstructive sleep apnea, chronic insomnia, restless legs syndrome, depression, and medication-related sleep disruption. Cognitive behavioral therapy for insomnia and evidence-based apnea treatment should precede unvalidated sleep supplements.

Stress, Social Connection, and Lessons From Blue Zones

Acute stress is adaptive; chronic uncontrollable stress produces allostatic load through repeated neuroendocrine, autonomic, metabolic, and immune activation [71]. Psychological stress has been associated with shorter telomeres, and longitudinal work suggests that greater cortisol reactivity may predict faster telomere attrition, although these biomarkers are not deterministic [72,73]. Disturbed sleep, inactivity, and social isolation can converge on chronic inflammation, which is linked to cardiometabolic disease, neurodegeneration, cancer, and frailty [74,75]. Stress management should therefore be concrete: treatment of anxiety or depression, physical activity, adequate recovery, time in nature, mindfulness or relaxation for willing patients, and reduction of occupational, financial, caregiving, or discrimination-related burdens where possible. Social connection is not a lifestyle accessory. Meta-analyses consistently associate stronger relationships with better survival and identify both loneliness and objective isolation as mortality risks, although observational confounding and bidirectionality remain [54,76,77]. Clinicians can screen for loneliness, bereavement, hearing impairment, mobility barriers, and caregiver strain, and connect patients with group activity, volunteering, community organizations, psychotherapy, or social services. Blue Zones are geographically defined populations reported to have exceptional longevity, including parts of Sardinia, Okinawa, Nicoya, and Ikaria. Age validation has been strongest in some historical cohorts, including central-eastern Sardinia, but demographic accuracy, migration, cohort change, selective storytelling, and commercialization complicate causal claims [78,79]. The traditional Okinawan pattern combined low energy density, high vegetable and legume intake, and historically low caloric intake, but later generations experienced substantial dietary and health transitions [80]. Recurring features, routine movement, minimally processed plant-rich diets, social integration, purpose, and low tobacco exposure, are plausible and align with independent evidence. Blue Zones should be viewed as natural experiments that generate hypotheses, not controlled trials proving a universal recipe. In particular, alcohol should not be recommended for longevity: potential observational signals do not outweigh cancer, liver, cardiovascular, addiction, injury, and interaction risks (Table 6).

**Table 6:** Practical interpretation of lifestyle strategies for healthy longevity VO_2_max: maximal oxygen consumption

Domain	Practical approach	Relevant outcomes	Qualification or caution
Aerobic base [51,55-58]	Accumulate regular moderate-intensity activity; Zone 2 training can be utilized to build tolerable volume	Cardiorespiratory fitness, metabolic health, endurance, blood pressure	Heart-rate zones are method-dependent; progress according to symptoms, underlying disease, and recovery capacity
Vigorous fitness [51,55,56,61]	Incorporate interval training or vigorous-intensity exercise when appropriate and medically safe	VO2max, functional exercise capacity, time-efficient fitness gains	Not a substitute for total daily movement; medical screening and gradual progression may be indicated
Strength, power, and balance [51,59,60]	Train major muscle groups regularly; integrate progressive balance and power exercises suited to individual capacity	Muscle mass, muscular strength, bone loading, glucose disposal, fall prevention, functional independence	Ensure protein and energy sufficiency; account for proper technique, pain, frailty status, and orthopedic limitations
Dietary pattern [50,52,80]	Emphasize minimally processed plant-forward foods, unsaturated fats, dietary fiber, and adequate high-quality protein	Cardiometabolic risk reduction, healthy body composition, microbiome diversity, micronutrient adequacy	Cultural acceptability, economic accessibility, disease-specific dietary restrictions, and sarcopenia risk must guide planning
Energy and meal timing [23,62-66]	Maintain sustainable energy balance; consider earlier, consistent daytime eating windows when feasible	Weight management, glycemic regulation, peripheral circadian alignment	Intermittent fasting is not proven to extend human lifespan; potentially contraindicated in frailty, pregnancy, or eating disorders
Sleep and circadian health [53,67-70]	Maintain consistent sleep-wake timing, prioritize morning light exposure and daytime activity, ensure ≥7 hours of sleep opportunity	Cognitive function, mood regulation, metabolic homeostasis, immune and vascular health	Clinically evaluate and treat insomnia, obstructive sleep apnea, restless legs syndrome, psychiatric illness, or drug-induced sleep disturbances
Stress and social connection [54,71-77]	Manage clinical mood/anxiety disorders, mitigate chronic allostatic stressors where possible, foster meaningful social relationships and community participation	Reduced allostatic load, improved regimen adherence, higher health-related quality of life, decreased cognitive and cardiovascular risk	Social determinants of health may constrain individual autonomy; proactive referral to social services, psychotherapy, and community networks may be required

To bridge the gap between preclinical hypotheses and verified clinical outcomes, candidate longevity interventions must be evaluated across an evidentiary spectrum ranging from molecular surrogate markers to hard mortality endpoints. Table 7 synthesizes pivotal randomized controlled trials and well-adjusted prospective cohorts to illustrate this translational divide. Notably, while dietary restriction and candidate nutraceuticals have demonstrated safety and selective target engagement, such as modest slowing of specific pace-of-aging algorithms in CALERIE or tolerability without cognitive improvement in SmartAge, they currently lack prospective evidence of extended disease-free survival or clinical rejuvenation in healthy humans. In sharp contrast, multi-domain lifestyle determinants, including objective cardiorespiratory fitness, adherence to minimally processed dietary patterns (e.g., PREDIMED), and day-to-day regularity of sleep timing, demonstrate substantial, dose-dependent associations with reduced all-cause and cardiovascular mortality across large, diverse human cohorts [23,42,52,55,67].

**Table 7:** Summary of landmark human trials and prospective cohorts evaluating longevity interventions BMI: body mass index; CI: confidence interval; CV: cardiovascular; EVOO: extra-virgin olive oil; HR: hazard ratio; MI: myocardial infarction; RCT: randomized controlled trial; SCD: subjective cognitive decline; UK: United Kingdom

Study/intervention	Design and population	Sample size (N)	Endpoint/biomarker assessed	Effect size (95% CI)/outcome	Covariates/statistical notes
CALERIE Trial (Waziry et al., 2023 [23])	2-year RCT; 25% caloric restriction in healthy non-obese adults	N = 220	DunedinPACE, PhenoAge, GrimAge clocks	DunedinPACE pace of aging slowed by 2%-3% (p = 0.005); no significant change on GrimAge or PhenoAge	Intention-to-treat comparison vs. ad libitum diet control.
PREDIMED Trial (Estruch et al., 2018 [52])	Multicenter RCT; Mediterranean diet in high CV risk adults	N = 7,447	Major adverse cardiovascular events (MI, stroke, CV death)	HR = 0.69 (95% CI, 0.53-0.91) (EVOO); HR = 0.72 (95% CI, 0.54-0.95) (nuts) vs. low-fat control	Multivariable Cox regression adjusted for age, sex, and baseline cardiovascular risk factors.
SmartAge Trial (Wirth et al., 2022 [42])	12-month double-blind RCT; oral spermidine in older adults with SCD	N = 100	Rey Auditory Verbal Learning Test (memory)	Spermidine vs. placebo memory change: between-group difference p = 0.28 (neutral primary outcome)	Intention-to-treat analysis; well-tolerated, but demonstrated lack of cognitive efficacy.
Cardiorespiratory Fitness Cohort (Mandsager et al., 2018 [55])	Retrospective cohort; treadmill stress testing	N = 122,007	All-cause mortality (median follow-up 8.4 years)	Elite vs. low fitness: HR = 0.20 (95% CI, 0.16-0.24); low vs. below-average: HR = 1.95 (95% CI, 1.86-2.04)	Adjusted for age, sex, BMI, smoking, hypertension, diabetes, and known coronary disease.
Sleep Regularity Cohort (Windred et al., 2024 [67])	Prospective cohort (UK Biobank wrist accelerometry)	N = 60,977	All-cause and cardiometabolic mortality	Most regular vs. least regular sleep quintile: all-cause mortality HR = 0.70 (95% CI, 0.65-0.77)	Adjusted for sleep duration, age, sex, physical activity, shift work, BMI, and comorbidities.

Discussion

Principal Findings

The biology of aging is modifiable in laboratory systems, but the evidentiary gradient is steep. Senolytics, rapamycin, metformin for non-diabetic aging, spermidine, NAD+ boosters, and epigenetic reprogramming remain investigational. No biomarker substitutes for survival, function, cognition, or quality of life. In contrast, exercise, muscle preservation, dietary quality, sleep health, tobacco avoidance, vaccination, cardiometabolic risk reduction, and social connection already have substantial human relevance [16,28,34,38,42]. The central translational problem is a mismatch of timescale and endpoint. Molecular interventions can alter signaling within hours and biomarkers within months, whereas prevention of multimorbidity or disability may require years to demonstrate. Trials therefore need intermediate outcomes, but surrogate endpoints can mislead when a marker is merely correlated with aging rather than causal or when treatment changes the marker through an unrelated pathway. A robust evidentiary ladder should move from replicated target engagement to multisystem functional improvement, acceptable long-term safety, and finally delayed clinical events. Biomarker panels are most useful when they complement, rather than displace, measures such as gait speed, strength, cognition, hospitalization, and independent living [7,8]. These domains interact. Exercise activates AMPK, improves mitochondrial quality, supports autophagy, lowers visceral adiposity and inflammation, and changes microbial metabolism. Diet supplies microbial substrates and influences mTOR and insulin signaling. Sleep and circadian timing regulate appetite, glucose handling, immunity, and training recovery. Social adversity can undermine every behavioral prescription. The most credible model is therefore multimodal and longitudinal, with molecular therapies added only when they outperform optimized prevention on meaningful outcomes [43,50,68]. This systems perspective changes how “response” should be defined. Improvement in one clock, cytokine, or metabolite may matter less than preserved strength or participation. An intervention that improves metabolic markers could still be unfavorable if it compromises immunity, healing, bone, muscle, or exercise adaptation. Precision longevity medicine should seek the best overall trajectory rather than the youngest value on every assay [7,8].

Strengths and Limitations

This review integrates molecular mechanisms with clinical implementation, explicitly distinguishing target engagement from patient benefit, and critically examines popular concepts such as Zone 2 training, biological-age reversal, microbiome optimization, and Blue Zones while acknowledging their useful components. Methodological constraints include reliance on PubMed/MEDLINE and English-language publications, omission of non-indexed regional cohorts, and the narrative design without protocol registration or formal risk-of-bias scoring (e.g., RoB 2, ROBINS-I). Additional limitations encompass the rapid evolution of the literature, heterogeneous definitions of longevity, sparse data from very old and globally diverse populations, and potential publication bias or commercial conflicts across biomarker, supplement, testing, and drug-development studies [6,22,32]. Evidence is further constrained by healthy-volunteer selection, short follow-up, inconsistent adverse-event reporting, and emphasis on average treatment effects, while modifiers such as sex, ancestry, baseline fitness, frailty, polypharmacy, and social context influence both efficacy and harm. Findings from long-lived animal models may not translate to humans, and observational longevity studies risk reverse causation, survivor bias, and inequitable access to care. Collectively, these limitations underscore the need for large pragmatic trials, pre-specified subgroup analyses, and linkage to long-term clinical outcomes rather than reliance on underpowered biomarker studies [7,8].

Implications for Practice

Clinical longevity care should begin with a conventional medical foundation and a functional baseline. Reasonable measures include blood pressure, lipids and glycemia when indicated; tobacco and alcohol exposure; vaccination and screening status; medication review; body composition interpreted cautiously; gait speed, chair rise or grip strength; aerobic capacity estimated or tested safely; cognition when clinically appropriate; sleep symptoms; nutrition security; and social connection. Follow-up should focus on trends and goals meaningful to the patient rather than maximizing every biomarker [10,51,53]. Experimental drugs should not displace treatments with proven outcome benefit. Any off-label consideration requires a defensible indication, known product quality, interaction review, monitoring plan, stop rules, adverse-event reporting, and explicit disclosure that lifespan benefit is unproven. Changes in methylation age, microbiome composition, NAD+ concentration, or inflammatory markers should be interpreted as exploratory unless linked prospectively to clinical benefit [16,34,38]. In practice, the highest-value sequence is to identify major modifiable risks, establish sustainable movement, nutrition, sleep, and social goals, treat disease according to guidelines, and reassess function over time. Only after this foundation is addressed should an experimental intervention be discussed, ideally within a registered trial. This ordering is scientifically conservative but clinically ambitious: established prevention can affect several organ systems at once and is immediately available, while the molecular pipeline continues to mature [5,10].

Ethical, Practical, and Implementation Considerations

Longevity interventions raise questions about ageism, autonomy, opportunity cost, and justice. Medicalizing normal aging can stigmatize older people, while dismissing preventable decline as “just aging” denies care. Ethical practice treats disease and modifiable risk, preserves capacity, and studies mechanisms without promising immortality [3,6]. Equity is central because education, income, housing, environment, food, healthcare, and social inclusion shape longevity. Socioeconomic position and race are also associated with differences in methylation-based pace-of-aging measures, reinforcing the need to interpret biomarkers in social context [81]. Boutique testing may widen gaps while producing less benefit than population measures. Trials should represent sex, ancestry, socioeconomic position, multimorbidity, and frailty, and measure affordability alongside efficacy [3,4]. Data governance is also relevant because biological-age scores, genomes, wearables, and microbiomes can reveal sensitive information. Patients should know how data are stored, reused, sold, and 19 interpreted; algorithms require calibration across populations; and direct-to-consumer results should not trigger treatment without clinical validation [22,81].

Future Directions and Recommendations

First, geroscience trials should use harmonized composites of incident disease, disability, cognition, physical function, and quality of life, supported by biomarkers rather than replaced by them. Pragmatic platform trials could compare combinations while detecting interactions and harms [7,30]. Second, biomarker qualification should establish analytical validity, biological validity, responsiveness, and, most importantly, whether treatment-induced change predicts clinical benefit. Cross-tissue and ancestry validation, pre-specified analysis, blinded laboratories, and replication are essential [8,22]. Third, molecular interventions need precision. Senescent-cell subtypes, tissue-specific mTOR modulation, mitophagy flux, microbial function, and NAD+ compartmentalization are more informative than indiscriminate pathway manipulation. Dosing should account for intermittent versus continuous exposure and for differences by age, sex, frailty, and disease [11,12,27]. Finally, health systems should make the proven components of longevity easier: tobacco cessation, vaccination, resistance and aerobic exercise programs, treatment of hypertension and sleep disorders, access to nutritious food, hearing and vision care, fall prevention, and social prescribing. The field will have succeeded if added years are healthier and more equitable, not merely if a laboratory estimate becomes younger [3,4].

## Conclusions

Longevity science has progressed from descriptive theories to a coherent network of molecular hallmarks, quantifiable biological-age measures, and early translational trials. However, it has not yet produced a pharmacological intervention proven to extend healthy lifespan in humans. The evidentiary gap between laboratory lifespan extension and clinical benefit underscores that surrogate biomarkers and epigenetic clocks cannot substitute for hard functional outcomes or disease-free survival. The most defensible clinical paradigm remains a layered one: prioritize scalable lifestyle pillars, notably cardiorespiratory fitness, muscular strength, nutritional quality, and restorative sleep, while reserving investigational geroprotectors for registered clinical trials. Responsible longevity medicine replaces speculative claims of "anti-aging" with a pragmatic public-health objective: the equitable compression of morbidity and the preservation of intrinsic capacity, independence, and quality of life.
